# Intensity Modulated Radiation Therapy for Retroperitoneal Sarcoma: A Case for Dose Escalation and Organ at Risk Toxicity Reduction

**DOI:** 10.1080/13577140310001644751

**Published:** 2003

**Authors:** Mary Koshy, Jerome C. Landry, Joshua D. Lawson, Charles A. Staley, Natia Esiashvili, Rebecca Howell, Shahram Ghavidel, Lawrence W. Davis

**Affiliations:** Department of Radiation Oncology Emory Clinic and Emory University 1365 Clifton Road NE A1300 Atlanta GA 30322 USA

## Abstract

*Purpose:* Radiation therapy for retroperitoneal sarcoma remains challenging because of proximity to surrounding organs at risk (OAR). We report the use of intensity modulated radiation therapy (IMRT) in the treatment of retroperitoneal sarcomas to minimize dose to OAR while concurrently optimizing tumor dose coverage.

*Patients and methods:* From January 2000 to October 2002, 10 patients (average age 56 years) with retroperitoneal sarcoma and one with inguinal sarcoma were treated with radiation at Emory University. Prescription dose to the planning treatment volume (PTV) was commonly 50.4 at 1.8 Gy/fraction. CT simulation was used in each patient, three patients were treated with 3D-conformal treatment (3D-CRT), and the remaining eight received multi-leaf collimator-based (MLC) IMRT. IMRT treatment fields ranged from eight to 11 and average volume treated was 3498 cc. Optimal 3D-CRT plans were generated and compared with IMRT with respect to tumor coverage and OAR dose toxicity. Dose volume histograms were compared for both the 3D-CRT and IMRT plans.

*Results:* Mean dose to small bowel decreased from 36 Gy with 3D-CRT to 27 Gy using IMRT, and tumor coverage (V95) increased from 95.3% with 3D-CRT to 98.6% using IMRT. Maximum and minimum doses delivered to the PTV were significantly increased by 6 and 22%, respectively (*P* = 0.011, *P* = 0.055). Volume of small bowel receiving > 30Gy was significantly decreased from 63.5 to 43.1% with IMRT compared with conventional treatment (*P* = 0.043). Seven patients developed grade 2 nausea, three developed grade 2 diarrhea, one had grade 2 skin toxicity, and one patient developed grade 3 liver toxicity (RTOG toxicity scale). No other delayed toxicities related to radiation were observed. At a median follow-up of 58 weeks, there were no local recurrences and only one patient developed disease progression with distant metastasis in the liver.

*Conclusions:* IMRT for retroperitoneal sarcoma allowed enhanced tumor coverage and better sparing of dose to *critical normal structures* such as small bowel, liver, and kidney. Escalation of dose has a positive impact on local control for retroperitoneal sarcoma; IMRT may be an effective method to achieve this goal. We are evaluating preoperative dose escalation to 59.4 Gy.


*Sarcoma*, September/December 2003, *VOL. 7, NO. 3/4, 137–148*

Taylor & Francis healthsciences

ORIGINAL ARTICLE

### Intensity modulated radiation therapy for retroperitoneal sarcoma: a case for dose escalation and organ at risk toxicity reduction

MARY KOSHY, JEROME C. LANDRY, JOSHUA D. LAWSON, CHARLES A. STALEY, NATIA ESIASHVILI, REBECCA HOWELL, SHAHRAM GHAVIDEL & LAWRENCE W. DAVIS

*Department of Radiation Oncology, Emory Clinic and Emory University, Atlanta, GA 30322, USA*

**Abstract**

*Purpose:* Radiation therapy for retroperitoneal sarcoma remains challenging because of proximity to surrounding organs at risk (OAR). We report the use of intensity modulated radiation therapy (IMRT) in the treatment of retroperitoneal sarcomas to minimize dose to OAR while concurrently optimizing tumor dose coverage.

*Patients and methods:* From January 2000 to October 2002, 10 patients (average age 56 years) with retroperitoneal sarcoma and one with inguinal sarcoma were treated with radiation at Emory University. Prescription dose to the planning treatment volume (PTV) was commonly 50.4 at 1.8 Gy/fraction. CT simulation was used in each patient, three patients were treated with 3D-conformal treatment (3D-CRT), and the remaining eight received multi-leaf collimator-based (MLC) IMRT. IMRT treatment fields ranged from eight to 11 and average volume treated was 3498 cc. Optimal 3D-CRT plans were generated and compared with IMRT with respect to tumor coverage and OAR dose toxicity. Dose volume histograms were compared for both the 3D-CRT and IMRT plans.

*Results:* Mean dose to small bowel decreased from 36 Gy with 3D-CRT to 27 Gy using IMRT, and tumor coverage (V95) increased from 95.3% with 3D-CRT to 98.6% using IMRT. Maximum and minimum doses delivered to the PTV were significantly increased by 6 and 22%, respectively ($P = 0.011$, $P = 0.055$). Volume of small bowel receiving > 30 Gy was significantly decreased from 63.5 to 43.1% with IMRT compared with conventional treatment ($P = 0.043$). Seven patients developed grade 2 nausea, three developed grade 2 diarrhea, one had grade 2 skin toxicity, and one patient developed grade 3 liver toxicity (RTOG toxicity scale). No other delayed toxicities related to radiation were observed. At a median follow-up of 58 weeks, there were no local recurrences and only one patient developed disease progression with distant metastasis in the liver.

*Conclusions:* IMRT for retroperitoneal sarcoma allowed enhanced tumor coverage and better sparing of dose to *critical normal structures* such as small bowel, liver, and kidney. Escalation of dose has a positive impact on local control for retroperitoneal sarcoma; IMRT may be an effective method to achieve this goal. We are evaluating preoperative dose escalation to 59.4 Gy.

**Key words:** *IMRT, 3D-CRT, retroperitoneal sarcoma, VaRA, radiation therapy*

### Introduction

Intensity-modulated radiation therapy (IMRT) is a new and revolutionary method of radiation delivery based on the use of optimized non-uniform radiation beam intensities incident on the patient.[1] IMRT used in our department relies on an inverse planning system that employs computer-assisted optimization methods to determine the fluence intensities given to a specific tumor volume. By setting dose constraints to critical organs at risk (OAR) and tumor volume, dose conformality and OAR toxicity has been optimized. Local recurrence in retroperitoneal sarcoma is the primary cause of mortality in patients with this disease.[2–4] Retroperitoneal sarcoma has been responsive to radiation dose escalation,[5–7] yet efforts to achieve this with external beam radiation alone (EBRT) have been hampered by OAR toxicity. We report the use of IMRT as a means to minimize dose to OAR and concurrently maximize tumor dose coverage.

The therapeutic advantage of using IMRT with respect to toxicity profiles has been studied for a variety of different sites. Hong *et al.* recently reported the use of IMRT for whole abdomen radiation and found bone marrow dose reduction and improved tumor coverage when compared to traditional whole

We have no proprietary interest or conflicts of interest to disclose.

Correspondence to: Jerome C. Landry, MD, Emory Clinic Department of Radiation Oncology, 1365 Clifton Road NE A1300, Atlanta, GA 30322, USA. Tel.: +1-404-778-4051; Fax: +1-404-778-4139; E-mail: jerome@radonc.emory.org

1357-714X print/1369–1643 online © 2003 Taylor & Francis Ltd
DOI: 10.1080/13577140310001644751

abdomen treatment.[8] A five-field arrangement was used and the volume of pelvic bones receiving a dose > 21 Gy was reduced by 60% and tumor coverage improved by 11.8% with the use of IMRT. Clearly, the use of large fields, sometimes necessary for retroperitoneal sarcoma, does not preclude employment of IMRT. The presence of small bowel in the treatment field, as well as the close proximity of kidney and liver, have presented a limitation to dose escalation for tumors located in the abdomen. We reported on the use of preoperative IMRT in pancreatic cancer in which IMRT allowed for dose escalation to 61.2 Gy and resulted in reduced average dose to small bowel and a 10% reduction in volume of small bowel receiving > 50 Gy.[9] IMRT for head and neck cancers has resulted in a 2–30% incidence of late Grade 2 xerostomia in contrast to the 60–75% incidence reported with historical controls treated with 3D-conformal treatment (3D-CRT).[10,11] For prostate cancer, IMRT has resulted in a significant decrease in both acute and chronic rectal complications.[12]

Treatment of retroperitoneal sarcomas with radiation has been limited due to the close proximity of these tumors to small bowel, liver, and kidney. To avoid critically overdosing these organs at risk (OAR), the total dose delivered to the tumor is often compromised and, consequently, the risk of local recurrence is increased. Historically, these tumors have been treated with a 3–5-cm margin around the gross tumor volume (GTV) to include the anatomy of the involved tissues.[5,6,13] The Radiation Therapy Oncology Group (RTOG) in their currently open Phase II trial evaluating multimodality treatment for retroperitoneal sarcomas recommends a 5-cm circumferential margin, except in areas where sparing of dose to kidneys, liver, and spinal cord are required, in which a 3-cm margin may be allowed.[14] To treat with tighter margins than previously described in order to achieve dose escalation may potentially underdose the peritoneal cavity where the risk of local recurrence is the greatest. We believe that the use of IMRT and intent of dose escalation does not give one a mandate to compromise the margin that would normally be employed in the treatment of retroperitoneal sarcoma. The use of IMRT throughout treatment, from the beginning, allows for optimal dose minimization to OAR and maximization to tumor volume. Over the past 3 years, we have consistently employed IMRT with inverse treatment planning for the entire duration of preoperative radiation for patients with retroperitoneal sarcoma. Gross tumor volume, areas at high risk for local recurrence, and normal OAR are outlined on high quality CT images (2.5-mm slices) and a three-dimensional volumetric margin is obtained to produce a planning treatment volume (PTV).

Potential organ motion has been thought to compromise the benefits of IMRT; recent data indicate that the main effect of organ motion in IMRT is an averaging of the dose distribution, which is the same as for conventional treatment. Bortfeld *et al.* calculated and statistically analyzed the expected dose values and dose variances for volume elements of organs that move during the delivery of IMRT and found that the standard deviation was within 1% of the expected value for multi-leaf collimator (MLC) delivery.[15] To take into account potential organ motion, we employed an additional margin of 1 cm, which is built into our PTV margin to account for GTV movement with respiration. We previously defined the method of 3-D outlining areas of high risk as the volume at risk approach, or VaRA.[9] Here, we continue to use VaRA as an integral component of IMRT to ensure that tumor coverage is complete and minimal allowable dose is delivered to OAR. Although the kidneys and liver are dose-limiting structures when treating retroperitoneal sarcomas, the small bowel as an OAR poses the greatest challenge. Radiation doses beyond 45–50 Gy have been associated with small bowel obstruction; this is often the rate-limiting factor in dose escalation to a variety of tumors in the abdominal region.[16] We report our institutional experience with IMRT in the treatment of retroperitoneal sarcoma. We analyzed the benefits of IMRT with respect to the reduction of dose to critical OAR and enhanced tumor coverage. Dose–volume histograms of patients planned and treated with IMRT to 50.4 Gy were compared with 3D-CRT treatment plans to the same dose.

### Patients and methods

Between January and October 2002, 10 patients with retroperitoneal sarcomas and one patient with an inguinal sarcoma were treated with radiation in the Department of Radiation Oncology at Emory University School of Medicine. Two of the 11 patients had surgery at outside institutions while the remaining nine had surgery at our institution. All outside pathological specimens were reviewed internally. Average patient age was 56 years (range 34–82 years). Three patients presented with tumors < 10 cm, seven patients had tumors between 10 and 20 cm, and one patient had a tumor > 20 cm. Seven patients were female and four were male. Eight of the patients had primary tumors while the remaining three presented with recurrence of disease. Two of the 11 patients had pelvic involvement and nine of the eleven patients were treated with preoperative radiation followed by resection. Two patients were treated postoperatively. All patients were evaluated preoperatively by computerized tomography (CT) of the chest, abdomen, and pelvis and none had metastatic disease. Patient variables included age at diagnosis, sex, presentation status (primary versus recurrent), margin status, and extent of resection (Table 1). Tumor variables included size, location, histological subtype, histological grade, and stage (Table 1).

**Table 1.** *Clinicopathologic characteristics of 11 patients treated for retroperitoneal sarcoma*

| Pt. no. | Age | Sex | Size (cm) | Location | Histology | Grade | Prim/Re | TNM | Stage | Margins | Organ removal |
|---|---|---|---|---|---|---|---|---|---|---|---|
| 1 | 34.4 | Male | 4.5 × 3.6 × 1.7 | Inguinal | Myxoid liposarcoma | Low | R | T1b | I | Negative | N |
| 2 | 34.8 | Female | 10 × 10 × 8.5 | Retroper. | Embryonal rhabdomyosarcoma | High | P | T2b | III | Negative | Y |
| 3 | 69.3 | Female | 14 × 9.3 × 6.8 | Retroper. | Liposarcoma | High | R | T2b | III | Positive | Y |
| 4 | 76.9 | Male | 14.5 × 17.8 × 13.7 | Pelvic | Prostatic stromal sarcoma | High | P | T2b | III | Negative | Y |
| 5 | 65.6 | Female | 9.3 × 6.5 × 5.4 | Retroper. | Liposarcoma | Low | R | T2b | II | Positive | N |
| 6 | 56.0 | Female | 11 × 9 × 5 | Retroper. | Leiomyosarcoma | High | P | T2b | III | Negative | Y |
| 7 | 52.0 | Male | 17 × 19 × 25 | Retroper. | Liposarcoma | High | P | T2b | III | Positive | Y |
| 8 | 61.0 | Male | 23 × 13 × 19 | Retroper. | Liposarcoma | High | P | T2b | III | Positive | Y |
| 9 | 82 | Female | 12 × 15 × 20 | Retroper. | Liposarcoma | High | P | T2b | III | Negative | Y |
| 10 | 34 | Female | 4 × 5 × 6.5 | Retroper. | Leiomyosarcoma | High | P | T2b | III | Negative | N |
| 11 | 71 | Female | 18 × 11 × 10 | Retroper. | Leiomyosarcoma | High | P | T2b | III | Negative | Y |

For all 11 patients, we used the IMRT VaRA approach as well as 3D-CRT and dosimetric comparison. For visualization of small bowel, all patients were given three glasses of gastrograffin oral contrast and placed supine with arms above their head on a rigid foam cradle. Thirty minutes after drinking contrast, CT scans of the abdomen and pelvis were obtained. The AcQsim scanner (Picker, Cleveland, OH) was used in three patients and the General Electric (GE) light speed scanner (General Electric, Milwaukee, WI) in eight patients. The planning volume was scanned with 3.0-mm increments for the AcQsim and 2.5-mm increments for the GE scanner. These CT imaging studies were used to design our treatment plans. The GTV was defined as the visible gross tumor volume. The clinical tumor volume (CTV) was defined as expansion of the GTV to encompass potential microscopic spread of disease. The PTV for retroperitoneal sarcoma ultimately included the GTV plus a 5-cm margin in the superior and inferior dimensions and a 2-cm margin in the anterior/posterior and medial/lateral dimensions. The GTV, CTV, liver, kidneys, spinal cord, and small bowel were all outlined by the attending radiation oncologist. These contours were then sent to a 3-D treatment planning system (six patients were planned on CAD plan with Helios and five patients on Eclipse). Two plans were then generated including a 3D-CRT plan using a beams-eye view and an IMRT plan using inverse treatment planning with a sliding window approach, eight to 11 coplanar beams, and a 0.25 × 0.5-cm minimum beam resolution (Fig. 1). The PTV of both plans was designed to receive 100% uniformity of dose with the 95% isodose line encompassing the CTV + 2.5 cm and no more than + 110% inhomogeneity within the target volume. The 3D-CRT plan was typically composed of parallel, opposed oblique beams that employed multi-leaf blocking of portions of the kidneys, small bowel, and liver. For both IMRT and 3D-CRT plans, after 45 Gy the treatment margins were reduced to 2 cm around the GTV in all dimensions. Two patients did not receive a boost; one was treated with 3D-CRT to 45 Gy and dose escalation was not possible secondary to OAR toxicity and the other patient received re-irradiation to 36 Gy. The average volume treated was 3498 cc (1108–9040 cc). Eighteen-MV photons were used for the IMRT plans and 6-MV photons were used for the conventional plans, as these energies corresponded to the best dosing of these peritoneal-based tumors (because high energy IMRT beams came in from multiple directions, there was not a risk of superficial underdosing). Because of MLC restrictions and field widths larger than 15 cm, it was necessary to employ the technique of 'beam splitting'.[8,17] The GTV and OAR were all assigned an optimal dose, constraints, and priority. Table 2 and Figure 2 illustrate the various dose volume constraints that were placed. The PTV and GTV were usually assigned a constraint of 90% or greater while small bowel and other OAR were assigned a priority of 80% or greater. Isodose distributions, field arrangements, and DVHs were calculated for both plans. The prescription dose to the PTV was commonly 50.4 at 1.8 Gy/fraction with 45 Gy initially delivered to the PTV followed by a cone-down boost to the GTV with a 2.0-cm margin to 50.4 Gy. One patient was treated to 36 Gy at 1.2 Gy BID as a re-irradiation strategy and another to 59.4 Gy at 1.8 Gy qd postoperatively. CT simulation was used in each patient; three patients were treated with a 3-D conformal plan and the other eight received IMRT.

The acute toxicity of both 3-D CRT and IMRT was measured using the RTOG grading criteria. Using this scale, acute toxicity was assessed and recorded during each week of treatment and 3 weeks after radiation prior to surgery. Acute toxicity was also measured up to 3 months after surgery. Chronic treatment related toxicity was measured at each follow-up examination. RTOG scoring was used to measure both acute and chronic toxicities for all patients. Eleven patients were observed until March 2002. The median follow-up was 58 weeks.

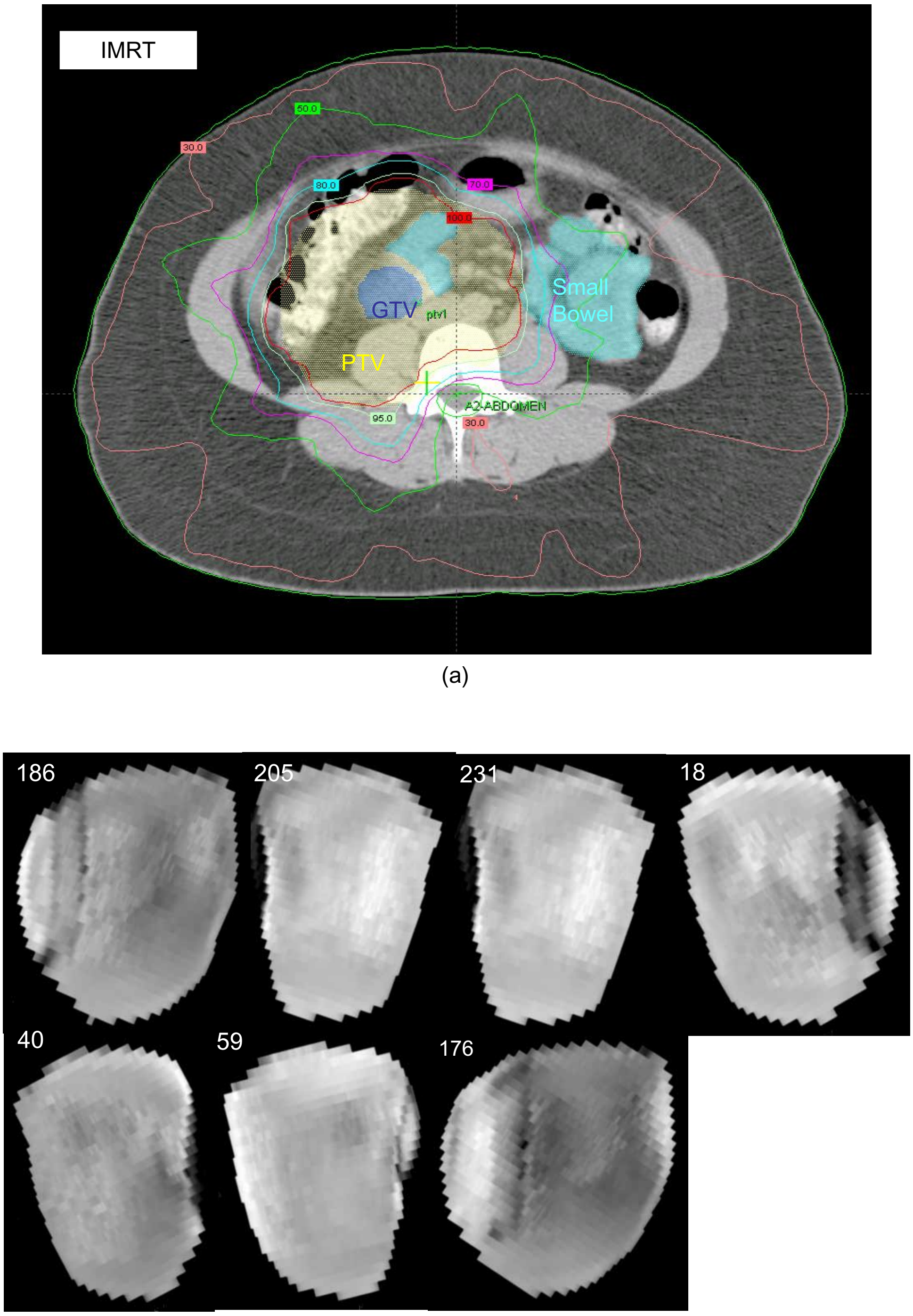

(a)

(b)

**Fig. 1.** *(a) Typical gantry angles for retroperitoneal sarcoma intensity modulated radiation therapy (IMRT); (b) intensity fluence maps with different gantry angles.*

Patients were followed with clinical examinations, chest X-ray, and CT scans of the abdomen/pelvis every 3 months after completion of therapy for 2 years and following this, every 6 months.

Complete resection was defined as resection of all gross disease with negative or microscopically positive margins. Local recurrence was defined as disease reoccurrence in the abdomen (retroperitoneum, peritoneal cavity, or intra-abdominal lymph nodes) while systemic recurrence was defined as recurrent disease in the liver or outside the abdomen.

Local recurrence was calculated on the basis of time from the date of surgery to the last follow-up examination. The significance of the DVH data by planning modality (3-D CRT versus IMRT) was determined by a paired two-sided *t*-test.

### Results

The beam's eye view, radiation field arrangements, and isodose comparisons between 3D-CRT and IMRT are illustrated in Figs. 3 and 4. Tumor coverage, tumor dose received, and OAR toxicity are further illustrated in comparative DVHs in Figs. 5 and 6. DVH data for all patients are summarized in Table 3. For the same dose constraints assigned to liver, small bowel, kidney, and PTV, IMRT resulted in improved coverage of the PTV and reduced dose to critical organs at risk. The difference was statistically significant for dose received to the small bowel and for the maximum and minimum dose received to the tumor volume. For the prescription dose to 50.4 Gy, both the maximum and minimum doses delivered to the PTV were significantly increased by 6 and 22% respectively ($P = 0.011$, $P = 0.055$) resulting in better dose distribution within the tumor volume. In addition, tumor coverage as measured by the V95 (volume receiving 95% of the dose) was improved from 95.3% with conventional treatment to 98.6% with IMRT, although this value did not reach statistical significance. The mean average dose to the small bowel decreased from 36 Gy with conventional 3-D conformal treatment to 27 Gy using IMRT. Furthermore, the mean dose to left kidney, liver, and spinal cord were all decreased with the use of IMRT. Although the difference in mean dose to the left kidney, liver, and spinal cord structures was not statistically significant due to the small sample size and large standard deviation, the overall trend favors IMRT. We believe it is possible to further decrease the dose to the aforementioned critical structures with IMRT, and this is being actively evaluated in our department.

**Table 2.** *IMRT inverse treatment planning algorithm constraint template for retroperitoneal sarcoma*

| Structure | Volume (%) | IMRT constraint criteria |
|---|---|---|
| Planning treatment volume (PTV) | 100 | Prescription dose: 45–50.4<br>Minimum dose: 45 Gy<br>Priority: 90% |
| Gross tumor volume (GTV) | 100 | Prescription dose: 50.4<br>Minimum dose: 45 Gy<br>Priority: 90% |
| Small bowel | 100 | Maximum dose: 45 Gy |
| | 75 | Maximum dose: 48 Gy |
| | 50 | Maximum dose: 50 Gy |
| | 25 | Maximum dose: 55 Gy<br>Priority: 80% |
| Kidney | 100 | Maximum dose: 12 Gy |
| | 50 | Maximum dose: 15 Gy<br>Priority: 80% |
| Liver | 100 | Maximum dose: 30 Gy |
| | 50 | Maximum dose: 40 Gy<br>Priority: 80% |

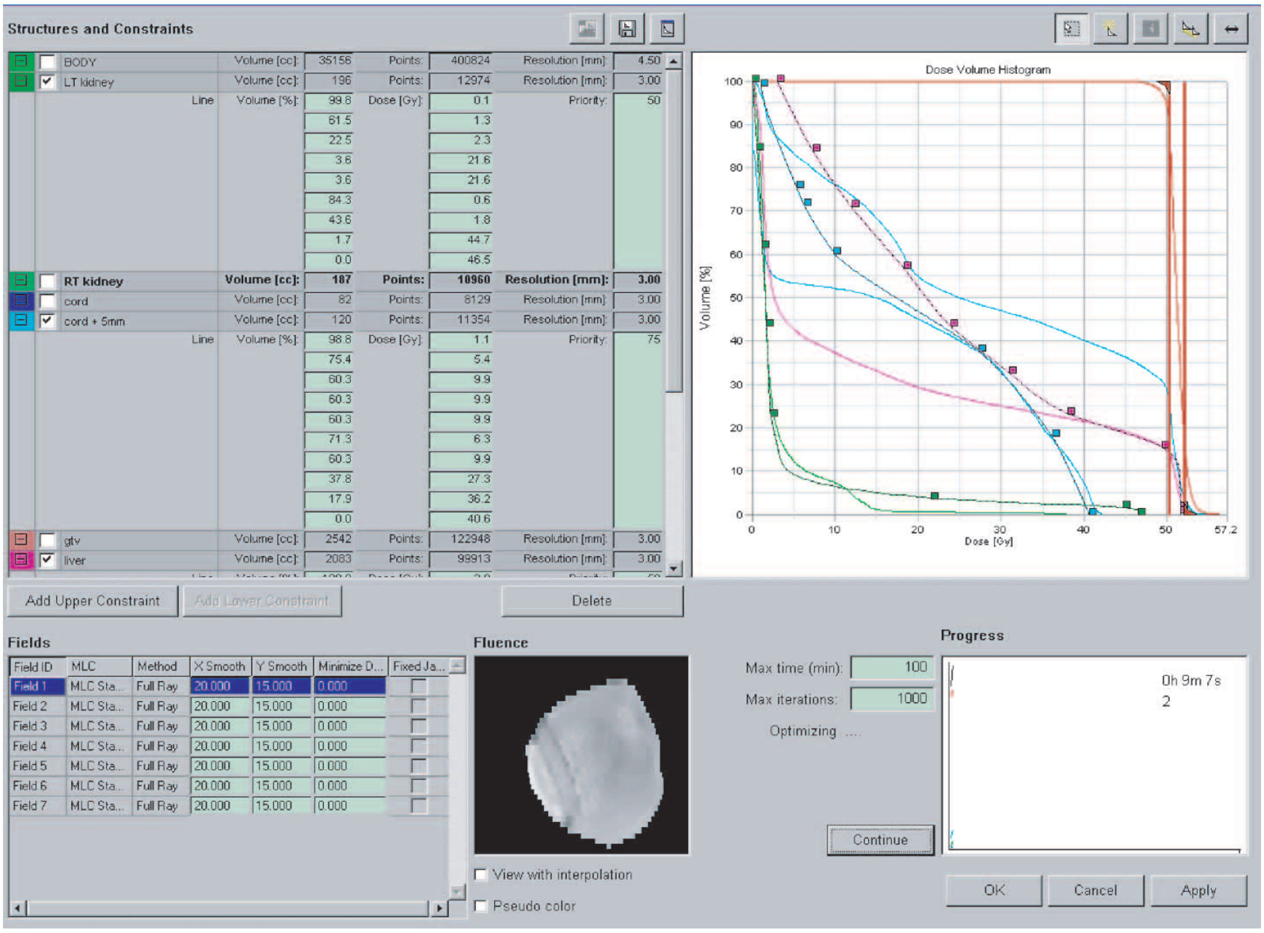

**Fig. 2.** *Illustration of dose prescription data for Eclipse planning system.*

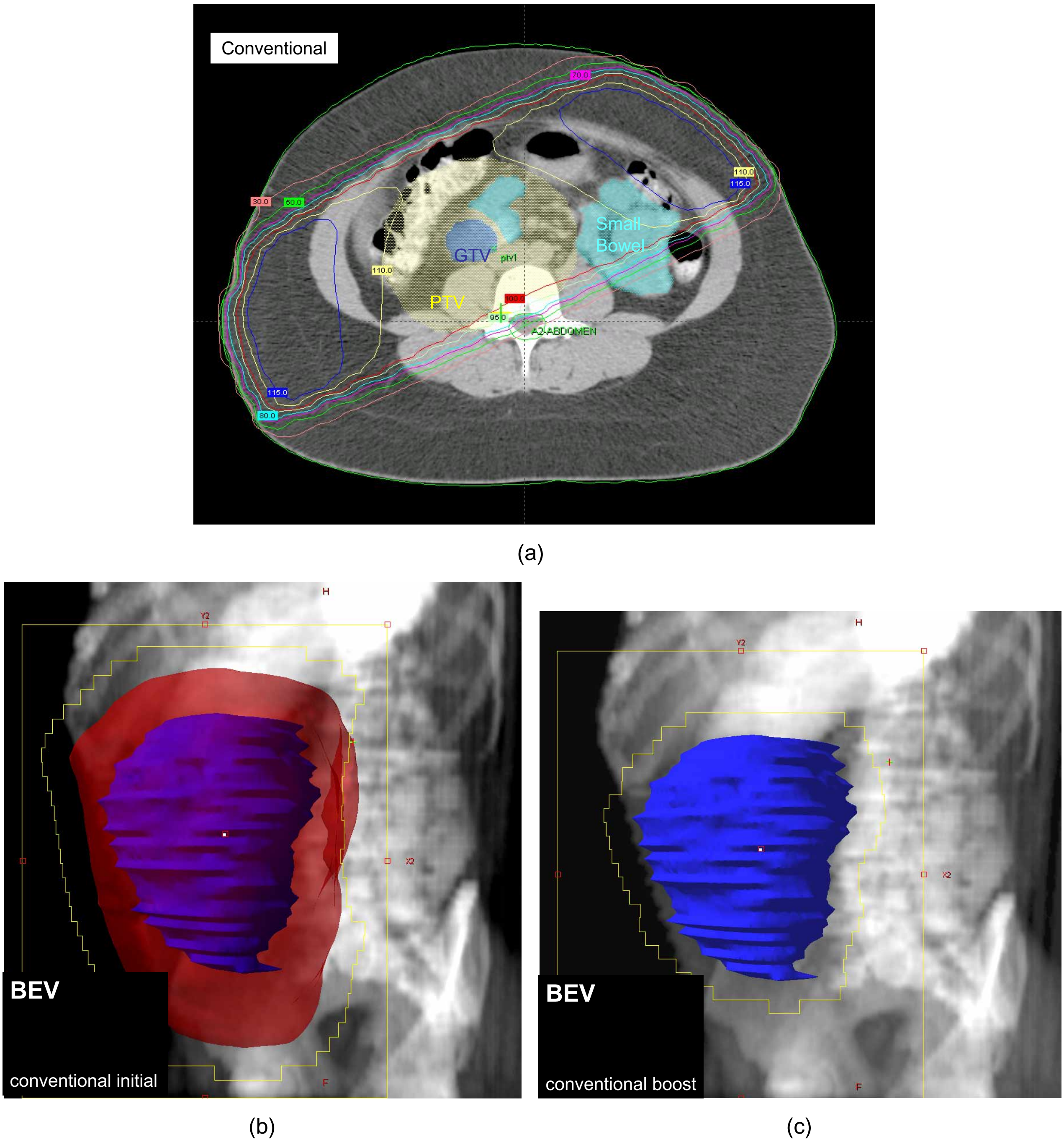

**Fig. 3.** *(a) Three-dimensional conformal radiation therapy (3D-CRT) isodose curve of composite field arrangement; (b) beam's eye view (BEV) of 3D-CRT initial plan for patient no. 8; (c) BEV of 3D-CRT boost plan for patient no. 8; PTV, planning tumor volume.*

The bladder and rectum, although included in our data, were only included in two patients, thereby precluding conclusive findings. The doses received by clinically significant volumes of small bowel, liver, and kidney with both IMRT and 3D-CRT were also analyzed (Table 4). The volume of small bowel receiving > 30 Gy was significantly decreased from 63.5 ± 25.2% (range 20–92%) to 43.1 ± 20.6% (range 20–92%) with IMRT compared with conventional treatment ($P = 0.043$). In addition, the median volume of small bowel that received a dose greater than 50 Gy and the dose delivered to one third of the bowel volume was reduced with IMRT. The median volume of small bowel that received a dose greater than 50 Gy was 8.8 ± 12.1% with IMRT compared to 23.5 ± 34.4% for 3D-CRT ($P = 0.073$). Figure 7 illustrates the clear advantage of IMRT over 3D-CRT with respect to dose delivered to the small bowel. The volume of left kidney that received a dose greater than 25 Gy decreased from 49 to 37% with the use of IMRT.

For patients with recurrent disease, recurrence varied from 3 to 6 years, and on average was 4.3 years. Eighty-two percent of tumors were high grade

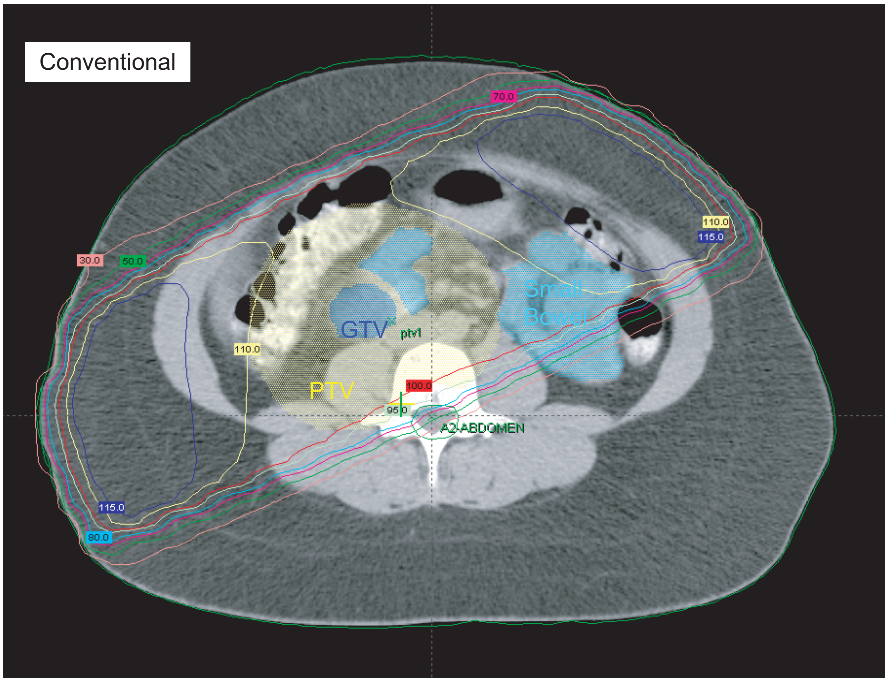

(a)

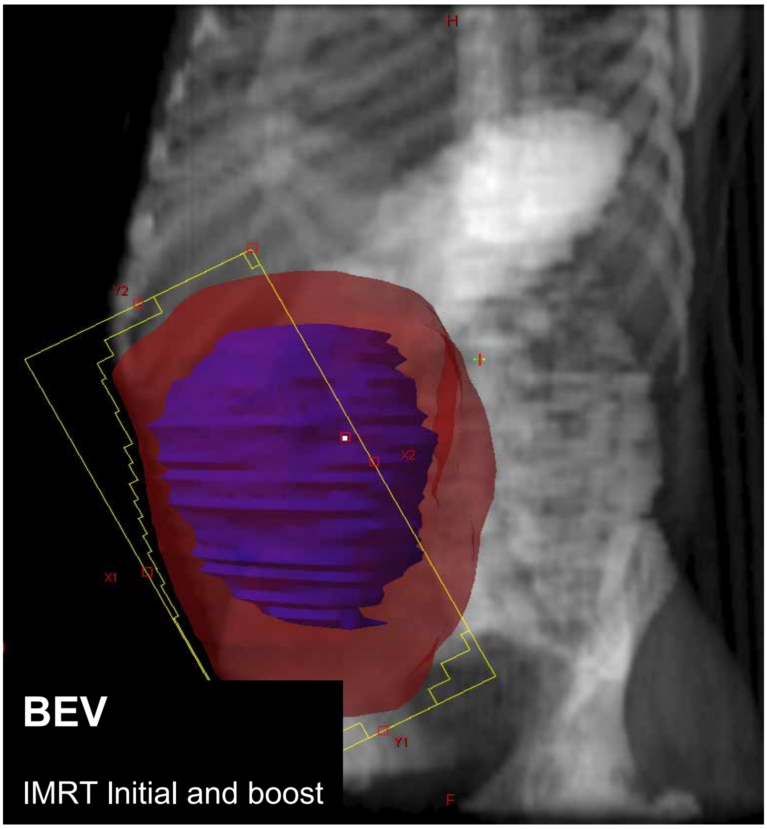

(b)

**Fig. 4.** *(a) IMRT isodose curve of composite field arrangement; (b) 59° split-field BEV of patient no. 8; PTV, planning tumor volume.*

histology while the remaining 18% were low grade histology. The majority of the resected tumors were liposarcoma and most patients presented with Stage III disease. Only two patients did not present with Stage III disease; one had Stage I, and one had Stage II tumor. All 11 patients had complete excision of gross tumor. On review of pathological specimens, four patients had microscopic positive margins and

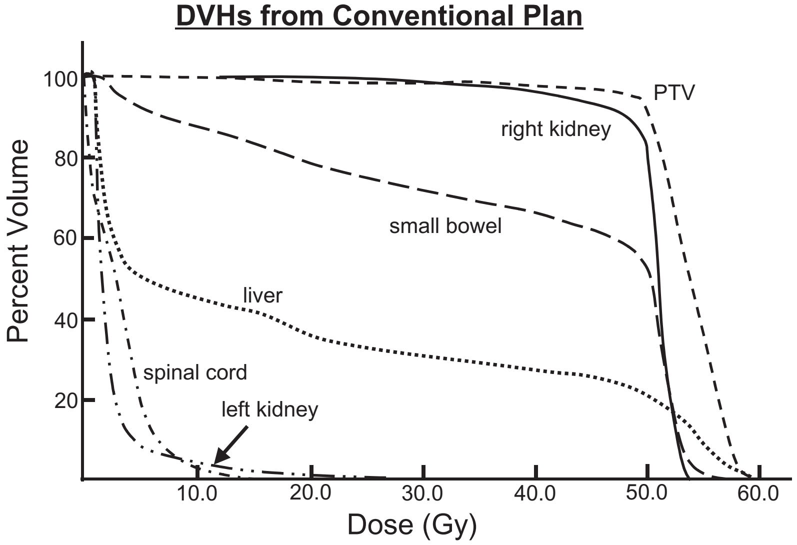

**Fig. 5.** *Composite dose volume histogram (DVH) for 3D-CRT for patient no. 8.*

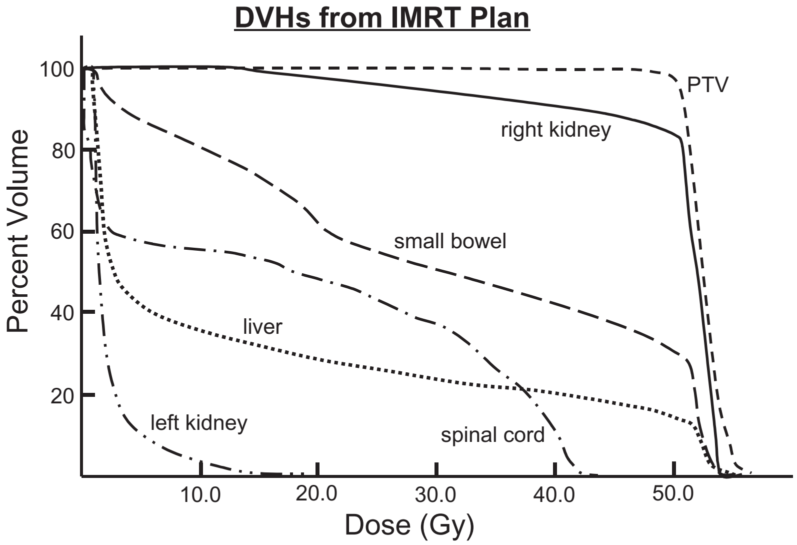

**Fig. 6.** *Composite DVH of IMRT for patient no. 8.*

the remaining seven patients had negative margins. A total of eight patients required some element of organ removal (defined as removal of the kidney, spleen, pancreas, adrenals, or colon) with nephrectomy the most common.

All patients were evaluated for toxicity using the RTOG toxicity scale (Table 5). The most common symptoms were nausea and vomiting and less frequently diarrhea. Seven patients developed grade 2 nausea, three developed grade 2 diarrhea, and one patient with primary groin involvement experienced grade 2 skin toxicity. One patient, who had extensive liver involvement and received 3D-CRT, developed grade 3 liver toxicity 6 months after his radiation and was hospitalized for management of ascites. This patient had approximately 85% of his liver involved with gross tumor and consequently 67% of the whole liver received 30 Gy, while 60% received 40 Gy with 3D-CRT. Currently, his ascites and hepatitis resolved and he remains free of disease recurrence. Other than this patient, there have been no other delayed toxicities related to radiation. No genitourinary (GU) or wound toxicities were observed and no treatment breaks were necessary. At a median follow-up of 58 weeks, there were no local recurrences and only one patient developed disease progression with distant metastasis in the liver (Table 6).

### Discussion

Retroperitoneal sarcomas account for 14% of all soft tissue sarcomas and 0.7% of all cancers diagnosed in the United States.[18] Surgical resection has been and remains the only curative modality for this disease.[19] Liposarcomas are the most common histological subtypes and make up about 50% of specimens in large series;[7,20,21] 62.5% of our specimens were of the liposarcoma subtype, in accord with this finding.

**Table 3.** *Summary dose–volume histogram data showing averages, ratio and* P *values for 11 patients with retroperitoneal sarcoma (all values based on 50.4 Gy prescription dose)*

| IMRT | Conventional (Gy) | Ratio (%) (Gy) | (IMRT/ Conv) | *P* value |
|---|---|---|---|---|
| PTV | | | | |
| D5 | 108 | 110 | | 0.117 |
| D50 | 103 | 100 | | 0.272 |
| MAX | 116 | 110 | | **0.011** |
| MIN | 62.3 | 40.1 | | **0.055** |
| MEAN | 102.1 | 102 | | 0.345 |
| V95 | 98.6 | 95.3 | | 0.312 |
| Left kidney | | | | |
| D5 | 69.5 | 92.3 | | 0.123 |
| D50 | 45.1 | 46.8 | | 0.472 |
| MAX | 87.1 | 97.8 | | 0.224 |
| MIN | 13.25 | 29.8 | | 0.190 |
| MEAN | 45.3 | 55.1 | | 0.320 |
| V95 | 21.5 | 35.6 | | 0.272 |
| Right kidney | | | | |
| D5 | 45.4 | 46.5 | | 0.478 |
| D50 | 29.1 | 34.9 | | 0.395 |
| MAX | 58.8 | 60.7 | | 0.468 |
| MIN | 10.2 | 15.6 | | 0.367 |
| MEAN | 29. | 29.2 | | 0.496 |
| Small bowel | | | | |
| D5 | 98.3 | 106.1 | 0.981 | 0.077 |
| D50 | 54.45 | 72.1 | 0.858 | 0.162 |
| MAX | 103.7 | 106.8 | 1.05 | 0.304 |
| MIN | 13.2 | 15.8 | 0.842 | 0.395 |
| MEAN | 56.9 | 70.8 | 0.870 | 0.133 |
| V95 | 22 | 35.1 | 0.633 | 0.190 |
| Liver | | | | |
| D5 | 87.1 | 108.5 | 0.822 | 0.066 |
| D50 | 40.1 | 43.5 | 0.950 | 0.449 |
| MAX | 95.7 | 112 | 0.896 | 0.080 |
| MIN | 3.8 | 10.1 | 0.264 | 0.243 |
| MEAN | 45.6 | 55.1 | 0.903 | 0.317 |
| V95 | 8 | 25.8 | 0.250 | 0.158 |
| Spinal cord | | | | |
| D5 | 61.5 | 86 | 0.697 | **0.046** |
| D50 | 38.5 | 45.3 | 0.682 | 0.341 |
| MAX | 81.9 | 91.1 | 0.825 | 0.073 |
| MIN | 1.2 | 1.12 | 1.00 | 0.467 |
| MEAN | 37.3 | 47.2 | 0.409 | 0.188 |
| V95 | 1.28 | 1.88 | 0.698 | 0.168 |
| Bladder | | | | |
| D5 | 100 | 104 | 0.962 | – |
| D50 | 76 | 101 | 0.752 | – |
| MAX | 102.5 | 104.5 | 0.981 | 0.152 |
| MIN | 55.4 | 49.2 | 1.13 | – |
| MEAN | 78.5 | 91.5 | 0.858 | 0.314 |
| V95 | 40 | 54.5 | 0.734 | 0.284 |
| Rectum | | | | |
| D5 | 74 | 94.5 | 0.783 | 0.390 |
| D50 | 54.5 | 75 | 0.727 | 0.291 |
| MAX | 77.5 | 100 | 0.775 | 0.375 |
| MIN | 57.5 | 53 | 1.08 | 0.459 |
| MEAN | 66.5 | 64 | 1.04 | 0.473 |
| V95 | 49.5 | 67.5 | 0.733 | 0.239 |

Abbreviations: D05, dose encompassing 5% of volume; D50, dose encompassing 50% of volume; V95, volume receiving 95% of the dose; Mean, mean dose; Max, maximum dose; Min, minimum dose; IMRT, intensity-modulated radiation therapy; Conv, conventional treatment.

Historically, rates of complete surgical resectability have varied from 38 to 65% with local recurrence rates as high as 70–90%.[2,21–24] Resectability in this study was 100% and may have been influenced by the delivery of preoperative radiation therapy. The vitality of a complete surgical resection has been documented in several studies and remains the single most important factor for survival.[23–26] Cody *et al.* in an evaluation of 158 patients noted a 5-year survival of 40% after complete excision but only 3% survival after an incomplete excision.[24] Because of the large tumor size at presentation and intimate involvement with adjacent organs, it is difficult to obtain resection with negative margins. Even with complete resection, local failure rates as high as 61–77% have been reported.[20,21,24] Unlike extremity sarcoma, local recurrence in retroperitoneal sarcoma is the primary cause of mortality in patients. Distant metastatic disease occurs in only one-third of patients and usually the liver is the first site of distant spread.[22] Clearly, this is a disease in which improvements in local control have the potential to significantly impact survival.

In an attempt to increase local control, post-operative radiation therapy has been given for retroperitoneal sarcomas. In a retrospective review of 198 patients, Heslin *et al.* noted patients who received postoperative radiation had a significantly reduced risk of local recurrence.[27] Local recurrence has proven to be insidious in this disease with many patients suffering recurrence after a 5-year disease-free interval; long-term follow-up is critical for evaluation of therapeutic intervention.[27]

Doses of 60–70 Gy have resulted in excellent local control of soft-tissue sarcoma of the extremities; extrapolating from this data it has been proposed that dose escalation may have a significant impact on local control of retroperitoneal sarcomas.[28] Tepper *et al.* reported on 17 patients with retroperitoneal sarcomas who were treated with external beam radiation therapy alone (shrinking field technique). In those patients who received a dose > 60 Gy, local control was 83%, compared to local control of only 18% for those treated to < 60 Gy.[7] At Princess Margaret Hospital, local infield failure rates tripled for patients with retroperitoneal sarcoma who received < 35 Gy compared to those who received > 35 Gy.[5] Fein *et al.*, in series of 21 patients with retroperitoneal sarcoma, noted at 2-year follow-up a local failure of 25% for those who received a dose > 55.2 Gy and 38% for those < 55.2 Gy.[6]

Most of the aforementioned reports involved the use of postoperative radiotherapy. In this study, we primarily used preoperative radiation, and believe that this approach is optimal for several reasons. The use of preoperative radiation may potentially reduce the risk of tumor seeding by shrinking the tumor and allowing for a more complete resection. With preoperative radiation, tumor mass is easily defined

**Table 4.** *Analysis of DVHs for small bowel, left kidney, and liver comparing IMRT and 3D-CRT treatment plans for patients with retroperitoneal sarcoma*

| | IMRT (Mean ± SD; range) | 3D-CRT (Mean ± SD; range) | *P* value |
|---|---|---|---|
| % of Small bowel > 30 Gy | 43.1 ± 20.6 (20–92) | 63.5 ± 25.2 (20–97) | **0.043** |
| % of Small bowel > 50 Gy | 8.8 ± 12.1 (0–31) | 23.5 ± 34.4 (0–85) | 0.073 |
| Dose to 33% of small bowel | 31.3 ± 7.9 (2–48) | 40.6 ± 11.5 (2–54) | 0.098 |
| % of Left kidney > 15 Gy | 50.3 ± 43.9 (1–100) | 55.1 ± 39.3 (3–100) | 0.422 |
| % of Left kidney > 25 Gy | 37.0 ± 40.6 (0–97) | 49.0 ± 41.9 (0–100) | 0.312 |
| Dose to 33% of left kidney | 27.0 ± 19.0 (2–47) | 28.7 ± 18.6 (2–47) | 0.442 |
| % of liver > 30 Gy | 33.3 ± 26.3 (1–60) | 49.6 ± 37.5 (13–100) | 0.201 |
| % of liver > 40 Gy | 26.8 ± 23.1 (0–50) | 46.0 ± 38.1 (11–99) | 0.158 |
| Dose to 33% of liver | 27.0 ± 19.0 (10–48) | 33.3 ± 19.2 (11–55) | 0.289 |

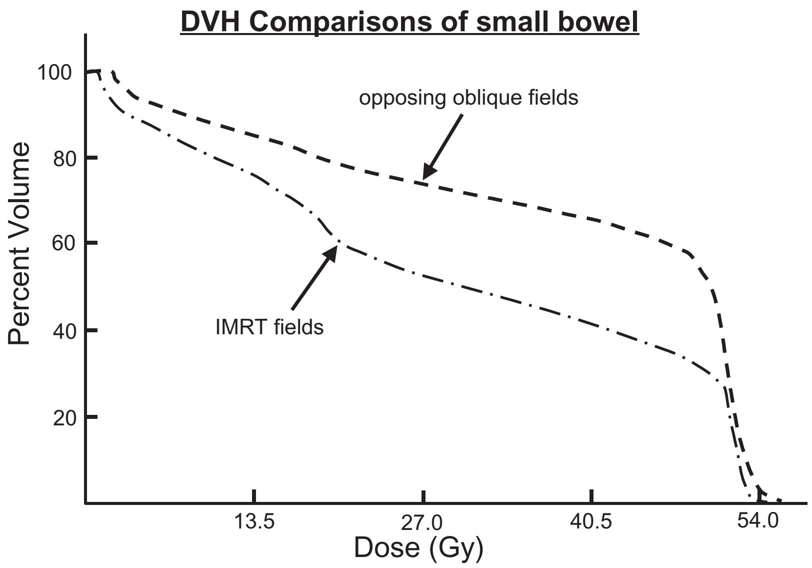

**Fig. 7.** *DVH comparison of small bowel for patient no. 8.*

**Table 5.** *Acute and chronic toxicity associated with IMRT for retroperitoneal sarcoma (RTOG toxicity scale)*

| | Skin | Upper GI | Lower GI | Liver | GU | Wound |
|---|---|---|---|---|---|---|
| Grade 1 | 0 | 0 | 0 | 0 | 0 | 0 |
| Grade 2 | 1 | 7 | 3 | 0 | 0 | 0 |
| Grade 3 | 0 | 0 | 0 | 1 | 0 | 0 |
| Grade 4 | 0 | 0 | 0 | 0 | 0 | 0 |

with CT/MRI and the risk of 'tumor miss' secondary to mobility in the postoperative abdomen is decreased. We do not employ IMRT for abdominal tumors in the postoperative setting, primarily because of lack of a precise and definable target and the increased risk of OAR displacement once the tumor has been removed. Treatment with radiation in the preoperative setting is beneficial for OAR because large retroperitoneal tumors often expand normal tissue out of the radiation field, thereby reducing exposure. Furthermore, extrapolating from data for extremity soft-tissue sarcoma, the use of preoperative radiation therapy may result in improved local control, as has been observed in the treatment of large extremity tumors.[28–30]

**Table 6.** *Local control and follow-up of patients with retroperitoneal sarcoma treated with IMRT/3D-CRT*

| Patient number | F/U since XRT | F/U since surgery | Local recurrence | Distant metastases |
|---|---|---|---|---|
| 1 | 14 | 17 | N | N |
| 2 | 11 | 8 | N | N |
| 3 | 7 | 4 | N | N |
| 4 | 24 | 21 | N | N |
| 5 | 23 | 21 | N | N |
| 6 | 26 | 23 | N | Y (liver) |
| 7* | 14 | 11 | N | N |
| 8* | 13 | 12 | N | N |
| 9 | 7 | 5 | N | N |
| 10 | 5 | 4 | N | N |
| 11* | 6 | 11 | N | N |

*Patients treated with 3D-CRT.

Other attempts to increase local control through dose escalation have included intraoperative radiation with electrons (IOERT) and high-dose-rate intraoperative radiation therapy (HDR-IORT). The only randomized trial conducted on IORT was done by the NCI in which 35 patients were randomized to IORT (20 Gy) + EBRT (35–40 Gy)

vs. 50–55 Gy with EBRT alone. At a median follow up of 8 years, patients who received IORT did not have a survival benefit but in-field local recurrence significantly decreased from 80% in the EBRT alone group to 40% in the IORT group.[31] In a recent update of the MGH experience, Gieschen *et al*. noted a trend toward improved local control and a significant survival difference in those patients who had IORT after preoperative external beam radiation for retroperitoneal sarcomas.[32] Alektiar *et al*., who employed HDR-IORT (12–15 Gy) and postoperative EBRT in a study of 32 patients, observed a local control rate at 5 years of 62%.[33] It is evident that IORT offers a clear local control advantage similar to that seen with dose escalation of 3D-CRT. Disadvantages of IORT include its decreased availability and gastrointestinal (GI) and neurological side effects. Sixty percent of patients who received IORT in the NCI trial had neurological complications of peripheral and sensory neuropathy, and a 6–16% risk of neurological side effects has been noted in other trials.[31–33] Gastrointestinal complications of 13–19% and fistula rates of 8–9% have also been reported with the use of IORT.[31–33]

We believe IMRT has the potential to increase local tumor control with fewer side effects than IORT. None of the patients treated with IMRT in our series developed late GI, GU, or wound toxicities associated with radiation and there were no acute toxicities above grade 2. Just as normal structures and organs would be shielded or displaced at the time of IORT, strict dose constraints can be placed with the use of IMRT, offering a similar therapeutic ratio. IMRT allows delivery of optimal dose to the tumor, while concurrently respecting the tolerance of other OAR. We are encouraged by the excellent toxicity profile and local control rate achieved thus far with the use of IMRT for retroperitoneal sarcomas. Longer follow-up is needed to confirm the benefit of IMRT in a disease that has a high propensity for local failure.

Patients with retroperitoneal sarcomas succumb to their disease process because of local recurrence that persists for years. Attempts to minimize local recurrence continue, and we think IMRT is a clinically feasible method to employ. Escalation of dose has a positive impact on local control for retroperitoneal sarcomas and IMRT provides a weapon to achieve this goal. The use of IMRT results in enhanced tumor coverage and reduced OAR toxicity, opening the door for dose escalation. We are presently evaluating preoperative dose escalation to 59.4 Gy with IMRT. Based on this study, we are encouraged by the excellent toxicity profile and local control in patients with retroperitoneal sarcomas treated with IMRT. Further investigation of the role of IMRT in multimodality management of retroperitoneal sarcomas is warranted.

### References

1. Intensity Modulated Radiation Therapy Collaborative Working Group. Intensity-modulated radiotherapy: current status and issues of interest. *Int J Radiat Oncol Biol Phys* 2001; 51: 880–914.
2. Zhang G, Chen KK, Manivel C, Fraley EE. Sarcomas of the retroperitoneum and genitourinary tract. *J Urol* 1989; 141: 1107–10.
3. Glenn J, Sindelar WF, Kinsella T, *et al.* Results of multimodality therapy of resectable soft-tissue sarcomas of the retroperitoneum. *Surgery* 1985; 97: 316–25.
4. Jaques DP, Coit DG, Hajdu SI, Brennan MF. Management of primary and recurrent soft-tissue sarcoma of the retroperitoneum. *Ann Surg* 1990; 212: 51–9.
5. Catton CN, O'Sullivan B, Kotwall C, *et al.* Outcome and prognosis in retroperitoneal soft tissue sarcoma. *Int J Radiat Oncol Biol Phys* 1994; 29: 1005–10.
6. Fein DA, Corn BW, Lanciano RM, *et al.* Management of retroperitoneal sarcomas: does dose escalation impact on locoregional control? *Int J Radiat Oncol Biol Phys* 1995; 31: 129–34.
7. Tepper JE, Suit HD, Wood WC, *et al.* Radiation therapy of retroperitoneal soft tissue sarcomas. *Int J Radiat Oncol Biol Phys* 1984; 10: 825–30.
8. Hong L, Alektiar K, Chui C, *et al.* IMRT of large fields: whole-abdomen irradiation. *Int J Radiat Oncol Biol Phys* 2002; 54: 278–89.
9. Landry JC, Yang GY, Ting JY, *et al.* Treatment of pancreatic cancer tumors with intensity-modulated radiation therapy (IMRT) using the volume at risk approach (VARA): employing dose-volume histogram (DVH) and normal tissue complication probability (NTCP) to evaluate small bowel toxicity. *Med Dosim* 2002; 27: 121–9.
10. Chao KS, Majhail N, Huang CJ, *et al.* Intensity-modulated radiation therapy reduces late salivary toxicity without compromising tumor control in patients with oropharyngeal carcinoma: a comparison with conventional techniques. *Radiother Oncol* 2001; 61: 275–80.
11. Lee N, Xia P, Quivey JM, *et al.* Intensity-modulated radiotherapy in the treatment of nasopharyngeal carcinoma: an update of the UCSF experience. *Int J Radiat Oncol Biol Phys* 2002; 53: 12–22.
12. Zelefsky MJ, Fuks Z, Happersett L, *et al.* Clinical experience with intensity-modulated radiation therapy in prostate cancer. *Radiother Oncol* 2000; 55: 241–9.
13. Willett CG, Suit HD, Tepper JE, *et al.* Intraoperative electron beam radiation therapy for retroperitoneal soft tissue sarcoma. *Cancer* 1991; 68: 278–83.
14. Radiation Therapy Oncology Group S-0124. A Phase II Study of Multimodality Therapy for Primary and Recurrent Retroperitoneal Sarcomas. Section 6: Radiation Technique. American College of Radiology 2001. Accessed at <www.rtog.com> on 10/10/02.
15. Bortfeld T, Jokivarsi K, Goitein M, *et al.* Effects of intra-fraction motion on IMRT dose delivery: statistical analysis and simulation. *Phys Med Biol* 2002; 47: 2203–20.
16. Coia LR, Myerson RJ, Tepper JE. Late effects of radiation therapy on the gastrointestinal tract. *Int J Radiat Oncol Biol Phys* 1995; 31: 1213–36.
17. Wu Q, Arnfield M, Tong S, *et al.* Dynamic splitting of large intensity-modulated fields. *Phys Med Biol* 2000; 45: 1731–40.
18. Brennan MF, Casper ES, Harrison LB, *et al.* The role of multimodality therapy in soft-tissue sarcoma. *Ann Surg* 1991; 214: 328–338.

19. Stoeckle E, Coindre JM, Bonvalot S, *et al*. Prognostic factors in retroperitoneal sarcoma: a multivariate analysis of a series of 165 patients of the French Cancer Center Federation Sarcoma Group. *Cancer* 2001; 92: 359–68.
20. van Doorn RC, Gallee MP, Hart AA, *et al*. Resectable retroperitoneal soft tissue sarcomas. The effect of extent of resection and postoperative radiation therapy on local tumor control. *Cancer* 1994; 73: 637–42.
21. McGrath PC, Neifeld JP, Lawrence W Jr, *et al*. Improved survival following complete excision of retroperitoneal sarcomas. *Ann Surg* 1984; 200: 200–4.
22. Storm FK, Mauve DM. Diagnosis and management of retroperitoneal soft-tissue sarcoma. *Ann Surg* 1991; 214: 2–10.
23. Jaques DP, Coit DG, Hajdu SI, Brennan MF. Management of primary and recurrent soft-tissue sarcoma of the retroperitoneum. *Ann Surg* 1990; 212: 51–9.
24. Cody HS 3rd, Turnbull AD, Fortner JG, Hajdu SI. The continuing challenge of retroperitoneal sarcomas. *Cancer* 1981; 47: 2147–52.
25. Kilkenny JW 3rd, Bland KI, Copeland EM 3rd. Retroperitoneal sarcoma: the University of Florida experience. *J Am Coll Surg* 1996; 182: 329–39.
26. Bevilacqua RG, Rogatko A, Hajdu SI, Brennan MF. Prognostic factors in primary retroperitoneal soft-tissue sarcomas. *Arch Surg* 1991; 126: 328–34.
27. Heslin MJ, Lewis JJ, Nadler E, *et al*. Prognostic factors associated with long-term survival for retroperitoneal sarcoma: implications for management. *J Clin Oncol* 1997; 15: 2832–9.
28. Suit HD, Mankin HJ, Wood WC, *et al*. Treatment of the patient with stage M0 soft tissue sarcoma. *J Clin Oncol* 1988; 6: 854–62.
29. Pollack A, Zagars GK, Goswitz MS, *et al*. Preoperative vs. postoperative radiotherapy in the treatment of soft tissue sarcomas: a matter of presentation. *Int J Radiat Oncol Biol Phys* 1998; 42: 563–72.
30. O'Sullivan B, Davis AM, Turcotte R, *et al*. Preoperative versus postoperative radiotherapy in soft-tissue sarcoma of the limbs: a randomised trial. *Lancet* 2002; 359: 2235–41.
31. Sindelar WF, Kinsella TJ, Chen PW, *et al*. Intraoperative radiotherapy in retroperitoneal sarcomas. Final results of a prospective, randomized, clinical trial. *Arch Surg* 1993; 128: 402–10.
32. Gieschen HL, Spiro IJ, Suit HD, *et al*. Long-term results of intraoperative electron beam radiotherapy for primary and recurrent retroperitoneal soft tissue sarcoma. *Int J Radiat Oncol Biol Phys* 2001; 50: 127–31.
33. Alektiar KM, Hu K, Anderson L, *et al*. High-dose-rate intraoperative radiation therapy (HDR-IORT) for retroperitoneal sarcomas. *Int J Radiat Oncol Biol Phys* 2000; 47: 157–63.
